# Usefulness of smartphone use among surgeons in clinical practice during the pandemic of COVID-19: a cross-sectional study

**DOI:** 10.1186/s12911-021-01563-1

**Published:** 2021-06-25

**Authors:** Ali Jasem Buabbas, Saad Aldousari, Adel K. Ayed, Maryam Safar, Omar Alkandari

**Affiliations:** 1 Department of Community Medicine and Behavioural Sciences, Faculty of Medicine, Kuwait University, P.O. Box 24923, Safat 13110, Jabriya, Kuwait; 2 Department of Surgery, Faculty of Medicine, Kuwait University, Jabriya, Kuwait; 3 Department of Urology, The University of Texas MD Anderson Cancer Center, Houston, TX USA; 4 Health Sciences Center, Faculty of Dentistry, Kuwait University, Jabriya, Kuwait; 5 Kuwait Institute for Medical Specializations, Yousif Al Roumi Street, Kuwait City, Kuwait; 6 Department of Pediatrics, Mubarak Al Kabeer Hospital, Jabriya, Kuwait

**Keywords:** Smartphone, Benefits, Surgeons, Clinical practice

## Abstract

**Background:**

With the magnitude and severity of the COVID-19 pandemic, the usual face-to-face consultation within a clinical setting is no longer feasible. Thus, this led to the need for alternate means to provide adequate patient care for surgical patients. This is where the role of smartphones comes into play, in which it is thus of paramount importance. This research study aimed to assess the usefulness of smartphones in surgical practice during COVID-19 pandemic.

**Methods:**

This cross-sectional study is based on a questionnaire distributed among surgeons in different levels of practice working at Kuwait governmental hospitals during the COVID-19 pandemic. The questionnaire was developed via Google Docs to collect data for the current study.

**Results:**

Out of 600 surgeons, 180 have responded to the questionnaire, giving a response rate of 30%. Of these, 42.8%, 85.5%, and 58.9% were aged between 35 and 44 years, were male, and Kuwaiti nationals, respectively. Almost all of the respondents (99.5%) were using smartphones for hospital-related work. The most common uses of the smartphones involved texting (70%), and viewing or taking images and videos using built-in cameras (60%) either in the emergency department, outpatient clinics, wards, or operating rooms. The majority of the respondents (88%) rated the use of smartphones in practice as important.

**Conclusion:**

This study revealed that using smartphones in surgical practice was prevalent among the respondent surgeons in Kuwait during the pandemic. The majority of them considered using smartphones in practice to be important, due to its benefits in facilitating doctor–doctor and patient–doctor communication, reviewing the literature, and making clinical decisions. Guidelines are required for proper and legal use of smartphone devices in medical practice. Accordingly, recommendations are suggested.

## Background

With the advancement of technology over the past decade, smartphones have become a ubiquitous tool, utilized by surgeons and patients alike. Owing to their high functionality and portability, these devices may be put to use in the world of medicine to aid patient care at a distance [1, 2]. The field of surgery is no exception. This field requires a prompt response to a patient’s needs, as well as good communication in consultations in order to provide adequate patient care [2]. During the COVID-19 pandemic, governments around the world, including the state of Kuwait, implemented curfews and social distancing as protective measures to minimize the risk of the viral infection spreading. Consequently, almost all healthcare services were suspended, except for urgent cases. Therefore, alternative approaches were welcomed to facilitate healthcare delivery during the pandemic, such as telehealth. In surgical practice, this involved the surgeon evaluating the patient’s case and providing a consultation at a distance, as well as remotely following up with the patient to detect any post-operative complications. Smartphones were effective for this particular situation [3].

Smartphones provide a means of rapid access to the Internet. This ultimately results in ease of access to email, enabling the rapid and easy transfer of patient data. Additionally, their built-in cameras and video-calling applications allow e-consultations and the communication of various aspects of cases between physicians [4]. Furthermore, a plethora of surgical applications are available for download from the App Store and Google Play, facilitating surgical education and providing informative sources for patients undergoing procedures [5], particularly during the pandemic [1]. Smartphones can improve pre-operative efficiency, surgical education, and post-operative care [4].

Telemedicine practice can be delivered via the smartphones that provide a means of e-consultations between patients and physicians, as well as the communication and transfer of data in different formats between physicians [1, 4]. A United Kingdom (UK) study about smartphone use among surgeons found that 79.3% of practising physicians expressed a willingness to use smartphone technology clinically [6]. Furthermore, the extant literature shows that, during the COVID-19 pandemic, smartphones and their applications played a significant role in several aspects of telehealth practice [7]. A United States (US) study of 1182 surgeons in Michigan used telehealth in various surgical specialties at higher rates during the COVID-19 pandemic than before [1].

In Kuwait, prior to the COVID-19 pandemic, smartphone use was prevalent among doctors [8], and the use of smartphone applications for hospital work depended on the individual’s preferences and willingness to integrate smart technology into their clinical practice. However, this was different during the pandemic, wherein smartphones played a key role in maintaining communication lines among medical staff and between medical staff and patients. Therefore, smartphones can be used as a telehealth tool to make significant improvements in healthcare delivery, and this occurred during the pandemic.

Owing to the COVID-19 pandemic’s magnitude and severity, usual face-to-face consultations within a clinical setting were limited to emergency cases, while social distancing and quarantine rules were enforced in many countries [7]. This led to physicians needing to find alternate means to provide surgical care and manage clinical cases from a distance [2, 7]. This was where smartphones came into play. On the basis of the literature, an investigation into the attitudes towards smartphone use, the utilization of smartphones, and the difficulties related to smartphone use among surgeons in the state of Kuwait is lacking, particularly in the context of a health crisis such as the COVID-19 pandemic.

This research study aimed to assess the usefulness of smartphones in surgical practice during the COVID-19 pandemic. The objectives were to: (1) explore the attitudes of surgeons with regard to smartphone use in the management of patients; (2) assess the utility of smartphones for consultations in surgical practice; and (3) identify the concerns of the surgeons regarding the use of smartphones in clinical practice. The outcomes from this study include (1) encouraging hospitals to support doctors in adopting smartphones officially in clinical practice and (2) providing suggestions to develop guidelines for the proper and legal use of smartphones in medical practice.

## Methods

### Study design, population, and sampling technique

This cross-sectional study used convenience sampling, which is a non-probability method [9], to approach all surgeons (n = 600) working at different levels in surgical practice in Kuwait governmental hospitals during the COVID-19 pandemic.

Healthcare in Kuwait is provided at three levels: primary care through healthcare centres, secondary care through general hospitals, and tertiary care through specialized centres. A patient requires a referral from a primary care or secondary care facility to obtain surgical care. In Kuwait, healthcare services are provided to citizens for free and to expatriates for small charges. There are six governorates in Kuwait, namely: Capital, Ahmadi, Farwaniyah, Mubarak Al-Kabeer, Hawally, and Jahra. One general hospital and a number of primary care centres are located within each governorate.

### Research instrument: a questionnaire

The questionnaire’ items were adopted from previous studies for use in this study [6, 10], with modifications made to suit research setting and objectives of the study. The questionnaire included four sections: (1) demographic data and background; (2) pattern of smartphone use in clinical practice; (3) usefulness of smartphones; and (4) privacy and security concerns regarding smartphone use. The questionnaire was developed using Google Forms for data collection and was in English only.

### Ethical approval

Ethical approval was obtained from the Research Committee at the Kuwait Ministry of Health prior to the distribution of the questionnaire (reference number: 2020/1479). An informed consent form was obtained from each participant who agreed to participate in completing the questionnaire.

### Data collection

The questionnaire was piloted with 10 surgeons to ensure the appropriateness and readability of its items. Slight modifications were made to the background information section after obtaining feedback from some respondents to better suit the study objectives. Later, the questionnaire was distributed electronically as a link to all surgeons through the WhatsApp application on their smartphones. This was done through the chairman of the department of surgery in each hospital. The data collection process took two months (June–July 2020).

### Statistical analysis

The data were analysed using the STATA statistical software package (STATA v.12, STATA Corporation, College Station, TX, USA). Categorical variables were expressed as numbers and percentages for the descriptive statistics. Evaluations of the possible associations between the different dependent variables and the demographic characteristics of the respondents were performed using a chi-square test or Fisher’s exact test as appropriate. Statistical significance was considered as *p* < 0.05.

## Results

### Demographic characteristics

Out of the 600 surgeons working in Kuwait governmental hospitals during the study period, 180 responded to the questionnaire, giving a response rate of 30%. Of these, the majority (42.8%) were 35–44 years old (Table 1). The vast majority (85.6%) were male, and 58.9% were Kuwaiti. Most of the respondents (56.1%) were board-certified surgeons. The remainder (43.9%) were either residents in training or assistant surgeons and registrars. No trainees were involved in this study. Only 7.2% of the respondents had contracted a COVID-19 infection requiring admission or quarantine. Most of the respondents worked at Jaber Al-Ahmad Al-Sabah Hospital (33.3%), which was the designated COVID-19 hospital in Kuwait, with a specific capacity of over 1000 beds.

**Table 1:** The participants’ demographic characteristics [n = 180]

Variable	N (%)
Age (years)	
25–34	50 (27.8)
35–44	77 (42.8)
45–54	35 (19.4)
≥ 55	18 (10.0)
Gender	
Male	154 (85.6)
Female	26 (14.4)
Nationality	
Kuwaiti	106 (58.9)
Non-Kuwaiti	74 (41.1)
Current position	
Trainee	0 (0)
Assistant surgeon/registrar	79 (43.9)
Board certified	101 (56.1)
History of admission or quarantine for confirmed or suspected COVID-19 infection	
Suspected	24 (13.3)
Confirmed	13 (7.2)
None	143 (79.5)
Hospital of work	
Jaber Al-Ahmad Al-Sabah	44 (33.3)
Mubarak Al-Kabeer	16 (12.1)
Farwaniyah	8 (6.1)
Amiri	9 (6.8)
Adan	5 (3.8)
Jahra	3 (2.3)
Ibn Sina	21 (15.9)
Sabah	1 (0.8)
Others	25 (18.9)
Computer skills rating	
Literate	9 (5)
Competent	124 (68.9)
Proficient	47 (26.1)

### Demographic data and associations with other variables

The results show that in terms of gender, the use of iPhones (as opposed to other smartphone brands) was statistically significantly higher among females compared to males (*p* = 0.009). However, no other gender-related associations were found.

In terms of age, a statistically significant value of *p* < 0.001 was found, as younger surgeons tended to have more medical applications compared to older surgeons (Table 2). When looking at surgeon level in practice, residents were more likely to own medical applications compared to board-certified surgeons (*p* = 0.038). In addition, surgeons with advanced computer skills were more likely to use medical applications on their smartphones (*p* < 0.001) and were more likely to use the built-in cameras of their devices for clinical photography and videos (*p* < 0.049).

**Table 2:** Demographic data and using smartphones with medical applications

Variable	Medical applications N (%)
Age (years)	
25–34	42 (84)*
35–44	46 (60)
45–54	17 (49)
≥ 55	5 (28)
Gender	
Male	91 (51)
Female	19 (73)
Nationality	
Kuwaiti	73 (69)*
Non-Kuwaiti	37 (50)
Current position	
Assistant surgeon/registrar	55 (70)*
Board certified	55 (54)
Computer skills rating	
Literate	6 (67)
Competent	73 (59)
Proficient	31 (66)

*p value < 0.05

### Use of smartphones in surgical practice

Almost all of the respondents (99.5%) used smartphones for hospital-related work, particularly for using Internet search engines to access relevant medical information. Sixty per cent of the surgeons used medical applications during their work, such as UpToDate, Medscape, MDCalc, and Touch Surgery. The majority of the respondents (88%) rated the use of smartphones in practice to be of importance, due to the benefits.

**Fig. 1:**
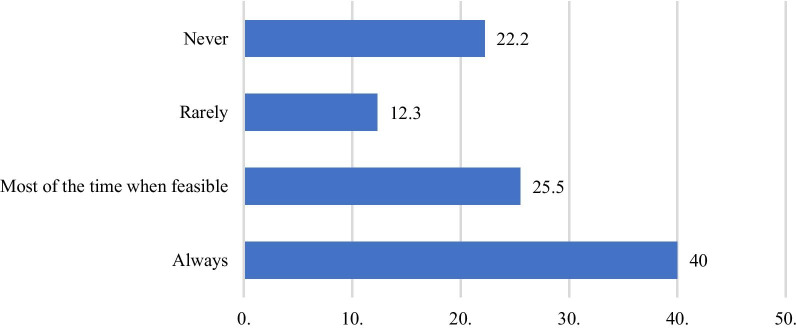
Percentages of surgeons who obtained consent from patients to use smartphones (%)

Regarding using smartphone technology for patient assessment, texting (70%) and viewing or taking images and videos using the built-in camera (60%) were the most common uses among the respondents, whether in the emergency department, outpatient clinic, ward, or operating room. The results show that almost half of the respondents (40%) always obtained consent from patients to use smartphones, and a quarter (25.5%) obtained it most of the time (Fig. 1).

**Fig. 2:**
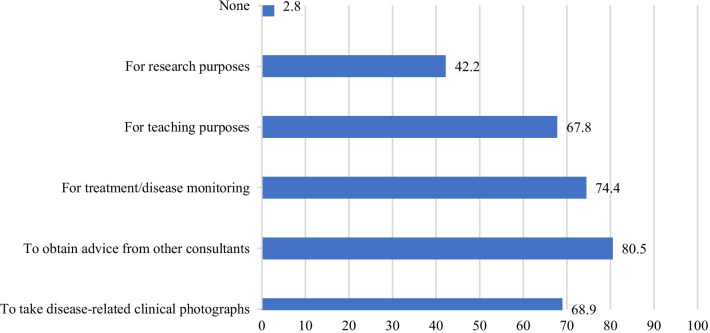
Percentages of surgeons who used smartphones’ built-in cameras for the respective purposes (%)

Regarding obtaining consent from patients, the results show that the majority of the respondents deemed verbal consent to be sufficient (55.6%). Only 23.9% obtained written consent, and a minority did not obtain consent, as they thought it was not needed (20.5%). Furthermore, 79.4% ensured patients’ awareness of their privacy rights regarding the clinicians’ use of smartphones in clinical practice, but 20.6% did not. In terms of the use of built-in cameras for photography, 80.5% said they used them to consult other consultants (Fig. 2).

A number of statistically significant associations were found with regard to the hospital of work. For example, surgeons at Jaber Al-Ahmad Al-Sabah Hospital tended to utilize the built-in cameras for clinical photography and videos (*p* < 0.000) and more often used smartphones to review patients’ X-ray images and laboratory test results (*p* = 0.014). In addition, the surgeons at this hospital more frequently used smartphones’ audio and visual functions for communication with other team members (*p* = 0.011). Compared to the surgeons working at other hospitals, no significant differences were found with respect to owning (*p* = 0.511) or using (*p* = 0.074) smartphones in general.

**Fig. 3:**
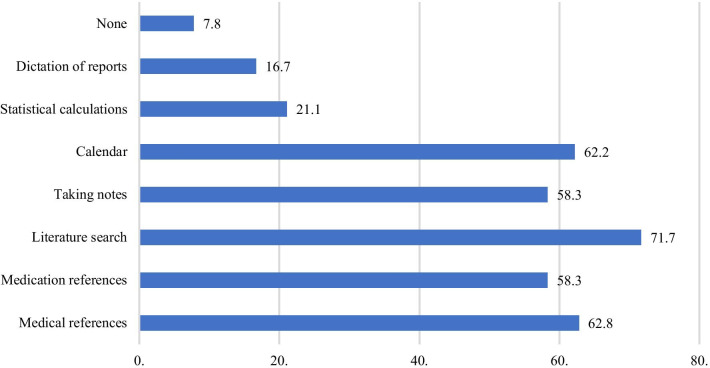
Percentages of surgeons who used smartphones for the respective non-communicative purposes (%)

Figure 3 shows that the majority of the surgeons used smartphones to search the Internet for medical literature (71.7%).

**Fig. 4:**
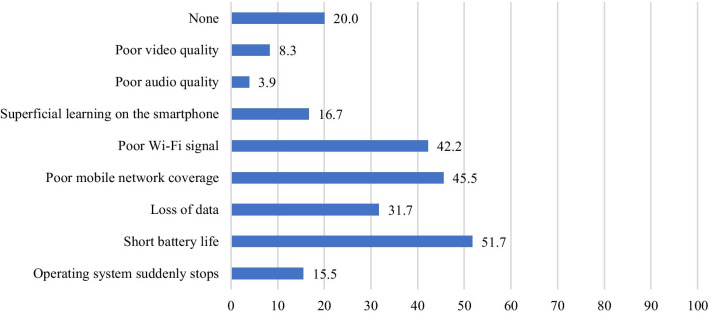
Percentages of surgeons who had experienced the respective difficulties and challenges in using smartphones during surgical practice (%)

Figure 4 shows the difficulties that the surgeons had faced during surgical practice in relation to smartphone use. A short battery life, poor Wi-Fi signal, and poor network coverage were the main difficulties faced.

Some of the surgeons (13%) shared their comments through the open comment box in the questionnaire. Thematic analysis was performed on these comments. Four surgeons reported the usefulness of smartphones in medical practice and considered it a gate to the World Wide Web (WWW). For instance, one surgeon had used the Google Translate application to help in communicating with a patient in the casualty department who could speak neither Arabic nor English. To quote: "it’s really a lifesaving tool”. Furthermore, five surgeons suggested that smartphones should be officially adopted in hospitals for clinical practice, stressing the need for regulations and training to ensure their proper use. In addition, four surgeons mentioned the demand for the use of smartphones for teleconsultation and telemedicine to improve communications for the purpose of patient care.

## Discussion

The findings of this study reveal the usefulness of smartphones among surgeons during the COVID-19 pandemic. The majority used their smartphones for surgical practice, utilizing various applications for hospital-related work. The results indicate the need for official regulations to ensure the proper and legal use of smartphones.

### Demographic data and associations with other variables

The findings reveal that in terms of gender, the use of smartphones was statistically significantly higher among females compared to males, but no other gender-related associations were found. This contrasts with the findings of other published studies, such as a UK study that found that male doctors were more likely to access the Internet using their smartphones to get medical information compared to female doctors [6]. However, a local study in Kuwait among dermatologists identified no gender differences with respect to smartphone use [8]. A similar finding was also found in a Saudi study on mobile phone use by medical residents [11].

The findings of the present study show that younger surgeons and residents in training tended to have more medical applications compared to older and board-certified surgeons, respectively. This is not unusual, as younger surgeons tend to be more oriented towards using the latest technology compared to older surgeons [8, 11, 12], and smartphones provide users with a wide spectrum of capabilities, including accessing media (e.g. videos and podcasts) and performing medical calculations (e.g. using MDCalc) [10, 13]. This finding was also found in a previous study, which reported that, compared to their seniors, junior doctors owned more medical applications [6, 8].

### Use of smartphones in surgical practice

The findings of the present study reveal that more than half of the respondents obtained verbal consent from their patients prior to using smartphones’ audio-visual functions for clinical use, and only a quarter obtained written consent. By comparison, in a local survey of dermatologists [8], 58.6% obtained written consent from their patients prior to using smartphones’ audio-visual functions for clinical use. The present study’s finding might be attributed to the emergent nature of surgical practice, which makes surgeons could not reserve written consent for surgical procedures and use verbal consent for smartphone use. It is very important for surgeons to consider updates in legal expectations in regards to document patients’ consent upon smartphone use in clinical practice [14]. This was apparent in the present study, as the majority of the surgeons ensured patients’ awareness of their privacy rights regarding the surgeons’ use of smartphones in clinical practice.

Clinical photography and video are significant uses of smartphone technology in surgical practice, and this was particularly evident during the COVID-19 pandemic. Previous studies have reported that the high quality of the images taken by built-in smartphone cameras increases the efficiency of taking and communicating images (as opposed to using a standard camera) [4, 8]. Smartphone cameras can be used to assess visible skin lesions, burns, blood smears, and alternative pathologies [4, 15–17]. However, another study reported the disadvantages of built-in smartphone cameras, which include compromised image quality and small screen sizes [4].

Under social distancing and quarantine rules and regulations to limit the spread of the virus, surgeons used virtual technology for consultations and communications with patients and other team members. Previous studies showed that smartphones are effective for following-up with the patients, wherein pictures and videos can be shared between patients and surgeons, accordingly medical decision can be made [3, 7]. Another study reported telehealth use among surgeons, in which there were not only follow-up patients, but also new patients were identified through this system [1]. A previous study reported that post-operative patients, particularly older patients, who had been discharged could remain at home and follow up with their surgeons using smartphone applications to assess symptoms and wound status [18]. The utilization of a telemedicine platform on a smartphone for the purpose of addressing wound concerns after day-case surgery results in a decline in the number of needless hospital visits [19, 20]. The findings of the present study support this, as the surgeons at Jaber Al-Ahmad Al-Sabah Hospital (the designated COVID-19 hospital) tended to utilize clinical photography and video on their smartphones more than the surgeons in other general hospitals in Kuwait did; they also more often used smartphone technology to review patients’ X-ray images and test results. Another explanation to support this finding is that Jaber Al-Ahmad Al-Sabah Hospital has a greater number of young board-certified surgeons compared to other general hospitals; accordingly, they were more likely to be skilled in computer and smartphone use, which was an association found in the present study.

The smartphone use difficulties experienced by the surgeons in the present study were similar to those identified in multiple studies in the literature, including a local study among dermatologists in Kuwait [8], which identified short battery life, poor Wi-Fi signal, poor network coverage, superficial learning, small screen size, errors in data input, and device viruses [6, 21–23]. However, a number of hospitals now have free Wi-Fi, allowing easy access to the Internet [6]. The provision of high-security Wi-Fi, as well as hardware and software firewalls, has been recommended in order to regulate smartphone usage within the clinical scenario [10]. In this context, data protection was the main concern mentioned by patients who utilized their smartphones for tele-follow-up after surgery [18]. The need for the provision of Wi-Fi was further endorsed by a study in Kuwait [8]. Further concerns mentioned by other studies include the validity of the utilized online resources and the stigma associated with smartphone use, making the physicians appear unprofessional to patients and colleagues [6]. Other concerns include the fact that smartphones may act as a distraction during work, and they may distribute pathogens in the workplace [6, 10, 11, 23, 24].

### Limitations

This study had several limitations: (1) most of the respondents were male. This is because most surgeons in Kuwait are male, and this may have biased the results; (2) the non-probability sampling method and the low response rate might have introduced sampling bias and caused the sample to be non-representative; and (3) the study did not include surgeons working in private hospitals because the private sector has its own policies and regulations, which differ from those of the public sector. Thus, the findings cannot be generalized beyond the study sample.

## Conclusion

This study revealed that the use of smartphones in surgical practice was prevalent during the pandemic among the respondents, enabling them to avoid face-to-face contact with patients. The majority considered using smartphones in their practice to be important, due to the benefits and usefulness in teleconsultations (doctor–doctor), telecommunications (patient–doctor), reviewing the literature, and making clinical decisions. The main difficulties associated with smartphone use can be solved, such as providing a free and strong Wi-Fi connection.

Regulations and training are required for the optimal and official use of smartphones in medical practice to overcome the concerns mentioned by the surgeons. Accordingly, the following recommendations are suggested. (1) It is very important to give medical staff access to electronic patient records (EPRs) via smartphones, giving them a complete picture of a patient’s case in order to make an accurate clinical decision. (2) Guidelines should be developed to: (a) suggest high-quality security applications that can be installed on smartphones, in order to make them eligible for hospital-related work; (b) suggest a number of trusted smartphone applications that can be used for surgical or medical practice; (c) maintain the legal rights of patients and physicians alike; and (d) maintain patients’ privacy and information confidentiality. (3) Information technology support should be offered to train medical staff in the different functions of smartphones for clinical use, as well as to ensure technical requirements are in place, such as the Wi-Fi connection, a long battery life, etc. (4) The hospital management should play a crucial role in managing medical practice that is supported by smartphone use. In addition, it is important to follow the guidelines of the American Telemedicine Association (ATA) with regard to telemedicine practice and smartphone use, together with the ATA’s *Quick-Start Guide to Telehealth During a Health Crisis* [25]. (5) Awareness about the role of smart technology in health care is very important for both patients and medical staff, and it should be obligatory to receive the patient’s consent when using smartphones in clinical practice.

## Data Availability

The datasets used and/or analysed during the current study are available from the corresponding author on reasonable request.

## Abbreviations

WWW: World Wide Web
UK: United Kingdom
US: United States
EPRs: Electronic patient records
ATA: American Telemedicine Association
